# Potential of a novel brine-struvite-based growth medium for sustainable biomass and phycocyanin production by *Arthrospira platensis*

**DOI:** 10.3389/fbioe.2024.1466978

**Published:** 2024-10-02

**Authors:** Stephan S. W. Ende, Albert S. Beyer, Reham Ebaid, Mostafa Elshobary, Mafalda C. Almeida, Cynthia Couto, Kit W. Chew, Tamara Schwenkler, Joachim Henjes

**Affiliations:** ^1^ Aquaculture Research, AWI—Helmholtz Centre for Polar and Marine Research, Bremerhaven, Germany; ^2^ Botany and Microbiology Department, Faculty of Science, Tanta University, Tanta, Egypt; ^3^ Academy of Scientific Research and Technology (ASRT), Cairo, Egypt; ^4^ Laboratory of Ecology of Microorganisms Applied to Aquaculture - LEMAQUI, Institute of Oceanography, Federal University of Rio Grande - FURG, Av. Itália, Rio Grande, Brazil; ^5^ School of Chemistry, Chemical Engineering and Biotechnology, Nanyang Technological University, Singapore, Singapore

**Keywords:** circular economy, desalination, nutrient recycling, phycoremediation, sustainability

## Abstract

Nutrient recovery is crucial for sustainability as it helps to recycle valuable resources, reduce environmental pollution, and promote the efficient use of natural materials in various agricultural and industrial processes. The present study investigated the impact of using brine and struvite as sustainable nutrient sources on the growth and c-phycocyanin (C-PC) production by the cyanobacterium *Arthrospira platensis*. Three modified growth media were compared to the standard SAG-spirul medium under yellow-white light [YLT], and blue-white light [BLT]. In the modified medium BSI, a struvite solution was utilized to replace dipotassium phosphate, while diluted brine was used to replace NaCl and de-ionized H_2_O. For BSII, struvite and brine were used as in BSI, with elimination of the micronutrient from the solution. In BSIII, no other nutrient sources than bicarbonate-buffer were used in addition to struvite and brine. For each medium, *A. platensis* was cultivated and incubated under YLT or BLT till the stationary phase. The results showed that the combinations of brine and struvite did not have any significant negative impact on the growth rates in BSIII. However, adding struvite as a phosphorus source boosted C-PC production just as effectively as YLT, with boosting biomass yield, unlike when only BLT was used. In conclusion, the brine/struvite-based media resulted in high biomass productivity with higher C-PC yields, making it an ideal growth medium for commercial sustainable C-PC production.

## 1 Introduction

The microalgae industry is continuously seeking sustainable, non-mineral-based nutritional sources to lower production costs while enhancing overall sustainability. Multiple waste streams have been evaluated and are used currently worldwide in large-scale microalgae farms. Different waste streams showed high potential for microalgae cultivation, which include food waste (Kumar et al., 2022), municipal wastewater (Han et al., 2024), anaerobic digestate (Bauer et al., 2021), and waste glycerol (Xu et al., 2019). In addition, microalgae showed high growth capability on high salinity water, including brine, i.e., the discharge of desalination plants (Al Bazedi et al., 2023). In 2017, there were about 19,372 desalination plants globally, a collective capability of producing around 99.8 million m^3^ of freshwater per day (WaterWorld, 2017). Per each liter of produced fresh water, there are about 1.5 L of highly concentrated brine produced (Jones et al., 2019). However, a significant environmental concern arises from the brine generated as a byproduct of desalination processes. Brine disposal methods commonly include sewer discharge, evaporation ponds, surface water discharge, deep-well injection, and land application (Mickley, 2018). Several studies have evaluated the adverse environmental effects of brine disposal on marine ecosystems, groundwater, and soil quality. Potential environmental damage may include pH fluctuations, eutrophication, and increased levels of heavy metals in marine environments and plant/animal mortalities (Panagopoulos et al., 2019).

In order to meet the carbon footprint derivatives of the European Union, many European countries aim to produce green energy from hydrogen. Sustainability will largely depend on the use of seawater, which is available in unlimited quantities and does not compete with water supply for human consumption. However, using seawater for hydrogen production requires prior desalination. This amount will comprise a problem since there is no concept for disposal of the produced brine. This may become the bottleneck for the entire energy transformation plans in countries counting on green hydrogen energy. Thus, finding new alternatives to remove and valorize brine eco-friendly and cost-effectively are of great importance. A recent approach is to convert brine into valuable biomass through microalgae cultivation in brine-based growth media. Several species such as *Chlorella vulgaris*, *Scendesmus quadricauda*, *A. platensis, Nannochloropsis oculata,* and *Dunaliella tertiolecta* have successfully been grown on brine-based media (Al Bazedi et al., 2023). Moreover, some microalgae, when cultivated under salinity stress, enhance the production of valuable compounds such as pigments, lipids, carbohydrates, and other bioproducts. This opens possibilities not just to reduce the volume of rejected brine but also to reduce production costs associated with prices for micronutrient media (Osman et al., 2023).

The microalgae market in European countries can not currently compete with the low-cost systems used e.g., in Asia, which limits market applications. Cyanobacteria like *A. platensis* have a high tolerance to elevated salinity. For instance, some *A. platensis* strains showed slightly reduced growth at 600 mM NaCl (3.5% salinity, similar to seawater) (Liu et al., 2016). Experiments showed that content of 5%–15% seawater in the culture medium only causes a decrease of biomass yield by 15%–20% as compared with Zarrouk’s culture medium (Tambiev et al., 2011). *A. platensis* has a unique quality to detoxify (neutralize) or to chelate toxic minerals and heavy metals (Okamura and Aoyama, 1994), and is also capable of removing minerals from inland saline waters (Sandeep et al., 2013).

Another cost driver in microalgae production is the source of macronutrients, i.e., nitrogen, phosphorus, and potassium. In order to reduce such costs, sustainable resources have successfully been tested in recent years. It was shown that replacing P with struvite-P enhances c-phycocyanin (C-PC) production in *A. platensis,* without compromising growth and biomass yield (Beyer et al., 2023). Struvite is the crystalized form of the mineral magnesium-ammonium-phosphate (MAP) with the formula NH_4_MgPO_4_ 6H_2_O. Several waste streams can be exploited for the production of sustainable struvite, e.g., urban wastewater, industrial wastewater, manure, or livestock slurries (Huygens et al., 2019). As struvite is already included as a commercial fertilizer in the Registration, Evaluation, Authorisation and Restriction of Chemicals (REACH) EC No. 1907/2006 and in the 2023 amendment to the EU eco-regulation/inclusion, the use of it in culture media is of special interest for the creation of new formulated sustainable media (European Commission, 2023).

Growth media combining both brine and struvite as micro- and macronutrient replacements have, to the best of authors’ knowledge, never been reported. If successful, such a cost-effective fully sustainable resource combination would open the opportunity for microalgae producers to enter new markets. Therefore, this study aims to test whether media combined of brine and struvite (recovered from Agri industries) is a suitable application for *A. platensis* production, with a special focus on C-PC production. It was cultivated on several media combinations, i.e., with brine (as a supplement to NaCl, micronutrients, and freshwater), and struvite (recovered from cow urine to replace mainly mineral P). The impacts on microalgal growth and C-PC yield were compared to the control groups using the standard SAG growth medium.

## 2 Materials and methods

### 2.1 Microalgal strain and inoculum preparation

*A. platensis* (UTEX 2340) used in this experiment was obtained from the culture collection of algae at the University of Texas, United States. The strain was preserved in spirul SAG medium (Aiba and Ogawa, 1977) under photoautotrophic conditions. For upscaling from the stock cultures, one 2 L glass bottle was prepared as inoculum. Further, 1,500 mL of SAG medium were inoculated with about 400 mL of stock culture. Cultures were illuminated at 105 ± 5 μmol m^−2^ s^−1^ with 12:12 light/dark cycle, provided by a daylight fluorescent tube.

### 2.2 Brine source and struvite preparation

Brine was collected from a desalination plant located on the Island of Helgoland, Germany (brine salinity was at 63.75 ppt). A comprehensive elemental characterization of the brine, including micronutrient analysis, was conducted using Inductively Coupled Plasma (ICP) spectroscopy and is summarized in Table 1. This analysis includes a comparison with brine water from other reverse osmosis desalination plants to provide context for our specific brine composition. Previous literature tested that the preferential salinity for *A. platensis* growth was 200 mmol (11.68 ppt) (Liu et al., 2016). To match this value, a new brine solution was prepared by adding de-ionized water to the brine by a factor of 4.45:1, respectively.

**TABLE 1:** Elemental analysis of the desalination brine compared to other brine sources.

Elements	Concentration (mg L^−1^)	Desalination plant in Oman (mg L^−1^)	Desalination plant in the canary islands (mg L^−1^)
Phosphorus (P)	<0.250	-	<0.10–0.44
Potassium (K)	583	43.1–668	588–916
Calcium (Ca)	465	417–1,020	703–1,181
Magnesium (Mg)	1,570	260–1980	2,105–3,036
Sodium (Na)	13,600	1,670–15300	17,761–21,070
Iron (Fe)	0.0300	<0.05	<10
Manganese (Mn)	0.00300	<0.05–0.07	<2
Boron (B)	4.89	-	6.3–8.1
Silicon (Si)	0.122	-	<2.4–48.2
Aluminium (Al)	<0.150	-	3–15
Molybdenum (Mo)	0.0140	-	<200
Arsenic (As)	0.00200	-	<2
Lead (Pb)	<0.0100	-	<2
Chromium (Cr)	0.0100	<0.02	<2
Zinc (Zn)	0.167	<0.05	<2–25
Selenium (Se)	<0.00100	-	
Barium (Ba)	0.0130	-	9–194
Strontium (Sr)	8.98	11.4–28.2	18.8–22.1
Lithium (Li)	0.210	-	0.6–1.4
Vanadium (V)	0.00700	-	3–16
Ammonium-Nitrogen (NH₄-N)	0.080	-	
Nitrate-Nitrogen (NO₃-N)	<0.10	5.2–46.7	0.2–48.2
References	This study	Ahmed et al. (2001)	Rivero-Falcón et al. (2023)

Based on previous successful applications of the pre-treatment (Beyer et al., 2023), microwave treatment was applied on the struvite for the BS I/II/III culture media in order to disintegrate the struvite and liberate nutrients. To prepare the struvite solution, 0.35 g of struvite was dissolved in 17.5 mL of 0.8 M NaOH solution (containing 0.56 g NaOH). This solution was heated in a microwave at 460 W for 8 min and then filtered. The entire 17.5 mL of this filtered solution was added to 1 L of the culture medium.

### 2.3 Culture media

The culture media were prepared as shown in Table 1, using SAG-spirul recipe as a control. For all tested media, bicarbonate buffer was used to stabilize the pH at 9.5. In the modified media [BS I], struvite solution was utilized as a replacement for dipotassium phosphate in solution I, while diluted brine was used as a replacement for NaCl and de-ionized H_2_O in solution II. The same was applied for BS II, but additionally, the micronutrients were omitted from the solution, to see if the micronutrients present in brine are sufficient. In BS III, bicarbonate buffer, struvite, and brine were added, while all other nutrients were omitted (Table 2).

**TABLE 2:** Chemical components of the used culture media with their respective modifications. SAG was based on the spirulina medium recipe (SAG), with an identical chemical composition. For BS I struvite substrate was used as an alternative phosphate source, and brine as a NaCl and de-ionized water replacement. BS II includes struvite as an alternative to dipotassium sulphate, and brine as a NaCl, de-ionized water and micronutrient solution replacement. In BS III I struvite substrate was once again used as an alternative phosphate source and brine as a replacement for solution II.

Components	*SAG*	*BS I*	*BS II*	*BS III*
*Solution I*				
De-ionized H_2_O (mL)	500	500	500	500
NaHCO_3_ (g)	13.61	13.61	13.61	13.61
Na_2_CO_3_ (g)	4.03	4.03	4.03	4.03
K_2_HPO_4_ (g)	0.5	0	0	0
Struvite (g)	0	0.35	0.35	0.35
NaOH (g)	0	0.56	0.56	0.56
*Solution II*				
De-ionized H_2_O (mL)	500	0	0	0
NaNO_3_ (g)	2.5	2.5	2.5	0
K_2_SO_4_ (g)	1	1	1	0
NaCl (g)	1	0	0	0
Brine solution (mL)	0	500	500	500
MgSO_4_ * 7H_2_O (g)	0.2	0.2	0.2	0
CaCl_2_ * 2H_2_O (g)	0.04	0.04	0.04	0
FeSO_4_ * 7H_2_O (g)	0.01	0.01	0.01	0
EDTA (mL)	0.08	0.08	0.08	0
*Micronutrient Solution (mL)*	5	5	0	0
	Stock solution [g/L]	Applied solution		
ZnSO_4_ * 7H_2_O	1	1 mL		
MnSO_4_ * 4H_2_O	1	2 mL		
H_3_BO_3_	2	5 mL		
Co(NO_3_)_2_ * 6H_2_O	0.2	5 mL		
Na_2_MoO_4_ * 2H_2_O	0.2	5 mL		
CuSO_4_ * 5 H_2_O	0.005	1 mL		
De-ionized H_2_O	-	981 mL		
FeSO_4_ * 7 H_2_O	-	0.7 g		
EDTA	-	0.8 g		

### 2.4 Experimental setup

Cultivation of *A. platensis* in different media was carried out in 1 L Erlenmeyer flasks and incubated at 29°C ± 2°C. They were incubated under a yellow-white LED-light source (LED Aquaristik, Hövelhof, Germany) for the first experimental trial. For the second experiment, the flasks were incubated under a blue-white LED light source. The Yellow-WL treatment consisted of a combination of yellow LEDs (peak emission at 590 nm) and white light LEDs. The Blue-WL treatment used a combination of blue LEDs (peak emission at 450 nm) with the white LED. For both light treatments, the average light intensity was set to 105 ± 5 μmol photons m^−2^ s^−1^ at light: dark cycle of 14:10 h. For further analysis, 10 mL of each culture were taken under sterile conditions, twice a week, up to 20 days for the first experiment, and 28 days for the second experiment after reaching the exponential phase.

### 2.5 Measurements and analysis

#### 2.5.1 Photometric measurements

For the photometric measurements of the optical density, 2 mL culture were taken and the absorbance was screened between 450 nm and 750 nm using S50 UV/Vis Biochrom Libra spectrophotometer to determine the absorption peaks. Based on the screening, absorbance values at 480 nm (at the absorbance peak of beta-carotene) and 700 nm (absorption maximum of the Qy band of the bulk chlorophyll a in the photosystem I) were used (Koehne and Trissl, 1998), 615 nm and 652 nm for the calculation of c-phycocyanin (Safari et al., 2020) were then recorded. For each sample, their respective typical culture medium was used as a blank. To determine the maximum specific growth rate [μ_max_] over a period from day 0 to day 20, a sigmoidal fit was applied to the data as previously described.

#### 2.5.2 Dry weight measurement

For the calculation of the dry weight from freshly taken samples, a correlation formula between optical density and dry weight was determined. For this, 50 mL of *A. platensis* were taken to prepare a dilution series. The absorbance of *A. platensis* stock and different diluted cultures was measured at 700 nm. The dry weight was measured using glass microfiber filters (Whatman), with a 1.2 μm particle retention previously dried for 10 min at 100°C in a microwave oven (CEM GmbH, Kamp-Lintfort, Germany). A volume of 5 mL of each fresh sample of the dilution series were filtered, then the filters were dried again at 100°C until constant weight. The dry weight was measured and the correlation between absorption of chlorophyll a [OD 700] and dry weight [DW] was derived from Equation 1;

$$DW\left({gL}^{-1}\right)=0.012+0.55gL^{-1}OD700\left(AU\right)$$ (1)

The propagated error on the calculated dry weight was determined using Equation 2;

$$\sigma_{DW}\left({gL}^{-1}\right)=\sqrt{0.55^{2}\sigma_{OD700}^{2}\left(AU\right)+{OD700\left(AU\right)}^{2}0.028^{2}gL^{-1}+0.026^{2}}$$ (2)

#### 2.5.3 Biomass and *in-vivo* C-phycocyanin productivities

Biomass productivity (BP, g DW L^−1^ d^−1^) was calculated from the values of the dry weight at the early stage of the exponential growth phase (t_E_, d) and at the late stage of the exponential growth phase (t_L_, d) using Equation 3 (Abomohra et al., 2013);

$${BP}_{t_{L}-t_{E}}=\left({DW}_{t_{L}}-{DW}_{t_{E}}\right)\times {\left(t_{L}-t_{E}\right)}^{-1}$$ (3)

*In-vivo* phycocyanin estimation has emerged as a valuable tool for monitoring cyanophytes in water sources and treatment plants (McQuaid et al., 2011; Marion et al., 2012; Beyer et al., 2023). Using Bennett and Bogorad’s formula, *in-vivo* c-phycocyanin yield ( $c_{CPC}$ , mg mL^−1^) of *A. platensis* was determined from the absorbance at 615 nm (OD615), and 652 nm (OD652) using Equation 4 (Safari et al., 2020);

$$c_{CPC}=\left(OD615-0.474\times OD652\right)*5.34^{-1}$$ (4)

*In-vivo* C-phycocyanin productivity (CPC-P, mg L^−1^ d^−1^) was determined using the concentrations of c-phycocyanin (c, mg mL^−1^) at early stage (t_E_) and later stage (t_L_) of growth (Abomohra et al., 2013) using Equation 5;

$${CPC⁃P}_{t_{E}-t_{L}}=\left(c_{t_{L}}-c_{t_{E}}\right)\times {\left(t_{L}-t_{E}\right)}^{-1}$$ (5)

### 2.6 Statistical analysis

Pearson correlation was used to quantify the linear relationship between two continuous variables. In addition, one-way ANOVA was used to compare the means of the four treatment groups that have been formed based on a categorical variable (medium used) with a significance level set to *P* < 0.05. To determine which specific groups have significantly different means, Tukey’s Honestly Significant Difference (HSD) test was used.

## 3 Results

### 3.1 Cell density and maximum growth rate

The effect of different culture media under different light sources on *A. Platensis* cultivation was evaluated. In general, results showed insignificant differences (*P* > 0.05) in beta-carotene and chlorophyll-a between different applied media (SAG, BSI, BSII, and BSIII) and between the two studied light treatments. Only one exception can be recorded for the maximum growth rate in BSI incubated in yellow-white light (YLT), which showed μ_max_ of 0.20 days^−1^ due to the minor variance between the values at 480 nm of their respective cultures [var.: 1.98 E^−4^].

**FIGURE 1:**
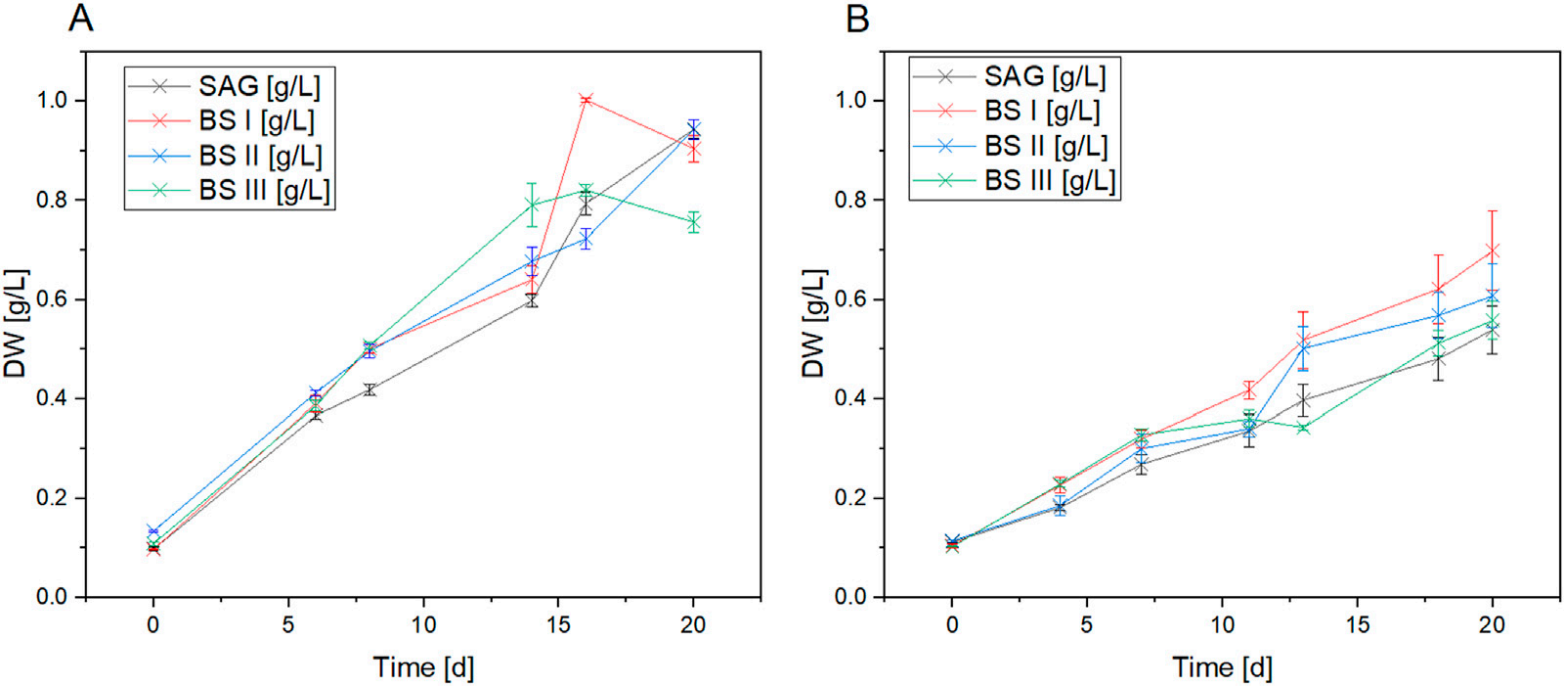
Dry weight data of *A. platensis* cultures grown in four culture media [SAG, BS I, BS II, BS III] under yellow-white light **(A)** and blue-white light **(B)** over an cultivation period of 20 days. Each data point shows the mean of the values derived from six dry weight values for each time point. n = 66 per treatment.

**FIGURE 2:**
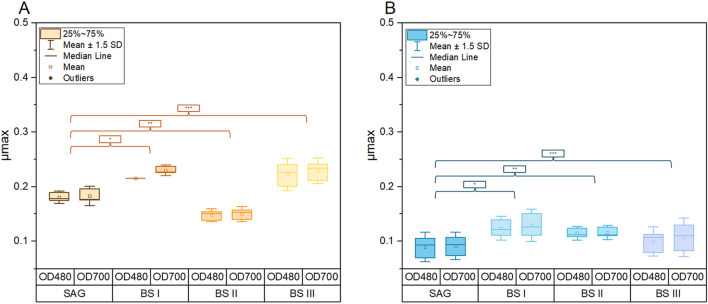
Comparison of maximum growth rates (μmax) of *A. platensis* cultivated in different culture media (SAG, BSI, BSII, BSIII) and different light conditions of yellow-white light **(A)** and blue-white light **(B)**. Each boxplot represents the specific generation times of three cultures per growth medium. The whiskers show the respective standard derivation with a coefficient of 1.5 *p*-values **(A)**: SAG/BSI (*): 480 nm = 0.02; 700 nm ≤ 0.001; SAG/BSII (**): 480 nm = 0.03; 700 nm = 0.03; SAG/BSIII (***): 480 nm = 0.01; 700 nm ≤ 0.001. *p*-values **(B)**: SAG/BSI (*): 480 nm = 0.09; 700 nm = 0.12; SAG/BSII (**): 480 nm = 0.024; 700 nm = 0.4; SAG/BSIII (***): 480 nm = 0.83; 700 nm = 0.7.

Figure 1 compares the growth of *A. platensis* in four different culture media (SAG, BS I, BS II, and BS III) under two light conditions (yellow-white and blue-white) over 20 days. The results clearly show that light quality significantly impacts growth, with yellow-white light promoting faster growth and higher biomass production across all media types compared to blue-white light. Under yellow-white light, the brine-struvite-based media, particularly BS II, outperform the standard SAG medium, reaching higher final biomass concentrations. BS II achieves the highest dry weight of around 1.0 g/L by day 15. In contrast, under blue-white light, growth is slower and more linear, with the SAG medium slightly outperforming the BS media. The stark difference in growth patterns between the two light conditions highlights the critical importance of light spectrum in *A. platensis* cultivation. Figure 2 summarizes the maximum growth rates. As shown in Figure 2A, a significant increase in the maximum growth rates for the YLT was recorded between the control with BSI and BS III (SAG/BSI: *P*-value at 480 nm = 0.02; *P*-value at 700 nm ≤ 0.001; SAG/BSIII: *P*-value at 480 nm = 0.01; *P*-value at 700 nm ≤ 0.001). The maximum growth rates between SAG to BSI and BSIII showed an increase by 39.2% (SAG/BSI) and 57.6% (SAG/BSIII). Between BSI and BSIII, the maximum growth rates showed no significant difference (BSIII/BSI: *P*-value at 480 nm = 0.88; *P*-value at 700 nm = 1.00). However, the growth in BSII was significantly lower than that in the control (SAG/BSII: *P*-value at 480 nm = 0.03; *P*-value at 700 nm = 0.03), with a decrease by 14.1% (SAG/BS II).

### 3.2 Biomass and C-phycocyanin productivity

**FIGURE 3:**
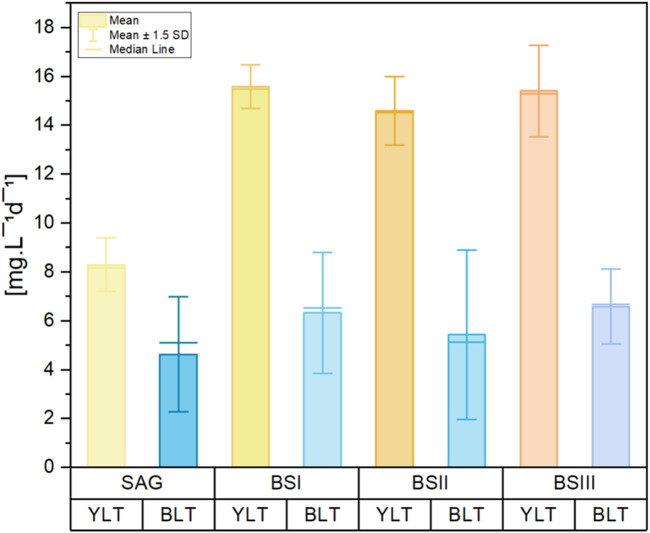
Bar graph of C-PC content per biomass per day [mg g^−1^d^−1^] of the cultures grown in different culture media (SAG, BS I, BS II, BS III) under yellow-white light (YLT) and blue-white light (BLT). Values were calculated with the data from an interval of early exponential phase to late exponential phase during the cultivation period. The whiskers show the respective standard error of the means. n = 3 per boxplot.

Table 3 illustrates the biomass productivity of *A. platensis* cultivated in different media at YLT and BLT. For YLT, biomass productivity significantly increased in BS I and BS III compared to the control, with *P*-values SAG/BSI = 0.02 and SAG/BSIII = 0.02. For BLT, ANOVA test showed no significant differences when comparing the four media, with F-value of 2.21 and *P*-value of 0.16. Regarding C-PC productivity in YLT, the values for SAG medium were significantly lower in comparison to all other tested media (BSI, BSII, BSIII) (Figure 3). The results demonstrate that yellow-white light consistently enhances C-PC productivity across all media compared to blue-white light. Under yellow-white light, all brine-struvite-based media (BS I, II, and III) outperform the standard SAG medium, with BS I showing the highest C-PC productivity, followed closely by BS III. This aligns with biomass productivity significantly increased in BS I and BS III compared to the control under yellow-white light. This suggests that the simplified, brine-struvite-based media are not only suitable for *A. platensis* growth but can actually enhance C-PC production, especially under optimal light conditions. Under blue-white light, C-PC productivity is generally lower and more consistent across media types, with BS III showing a slight advantage. For BLT, the production of c-phycocyanin showed no significant difference between different media (SAG, BSI, BSII, and BSIII), with F-value = 1.18 and *P*-value = 0.367.

**TABLE 3:** The biomass productivity during the exponential growth phase from day 0 to day 8 under yellow-white light (YLT) and from day 0 to day 7 under blue-white light (BLT) in different culture media (SAG, BSI, BSII and BSIII]. The standard error is shown, as well as the increase of each parameter in relation to the control group [SAG].

Light source	Parameter	Medium			
		SAG	BSI	BSII	BSIII
YLT	Biomass productivity [g L^−1^d^−1^]	0.04 ± 0.002	0.05 ± 0.001	0.045 ± 0.002	0.05 ± 0.001
	Increase to SAG (−) [%]	0	26.2	13.0	24.3
BLT	Biomass productivity [g L^−1^d^−1^]	0.01 ± 0.001	0.017 ± 0.00	0.0102 ± 0.003	0.018 ± 0.00
	Increase to SAG (−) [%]	0	76.0	2.2	81.5

The values for the C-PC content yield (expressed per biomass per day) are shown in Figure 3. For YLT, C-PC content yield was significantly higher in BS [I-III] culture media (*P*-value = 0.001). Contrary to YLT, C-PC content yield under BLT in BS [I-III] was not significantly higher compared to SAG (*P*-value = 0.46). Overall C-PC content yield was higher in the BTL, by 5.4% for BSI and 12.1% for BSIII, with a significant increase over SAG [by 407.5%; *P*-value = 0.01] and BS II [by 53.2%; *P*-value = 0.01]. All media of BLT showed significant differences with SAG of ZLT. However, with reduced growth in the BLT, biomass productivity was per average 70.4% lower than in the YLT.

## 4 Discussion

The present study is the first report proofing that *A. platensis* can be grown on local waste streams fully replacing mineral-based synthetic growth medium with struvite and brine. This would help the agri-industry sector to valorize and reduce waste streams from animal production, i.e., cow urine. In addition, it would provide the growing biohydrogen industry with a biological approach to valorize and reduce brine in the coming decades. On the other hand, the new suggested media will provide the microalgae industry with a near-zero cost growth medium at simultaneous high sustainability. This implies that brine can replace micronutrients coming from commercial micronutrient pre-mixes. It also implies that production can even be enhanced when commercial media and brine are used together.

Comparing the obtained maximum specific growth rate of *A. platensis* to values in literature, the present values using YLT in SAG showed 0.1808 days^−1^, which is higher than the stated values of the maximum specific growth rate of about 0.144 days^−1^ (Islam et al., 2003; Usharani et al., 2012). Results from the modified culture media BS I and BS III under YLT showed a significant increase in growth compared to the control group (SAG). Interestingly, using struvite and diluted brine as main nutrient sources in BS III resulted in a high maximum growth rate of 0.22 days^−1^, showing that both positively influence the growth of *A. platensis*. Interestingly, BS III, despite its minimal composition, performs nearly similar to BS I under yellow-white light. This indicates that *A. platensis* can effectively utilize the nutrients provided by struvite and brine, potentially reducing the need for additional nutrient supplementation. The slightly lower performance of BS II compared to BS I and BS III under yellow-white light might suggest some benefit from either the presence of micronutrients (as in BS I) or their complete absence (as in BS III), though this difference appears minor. The effectiveness of BS III can be attributed to the complementary nutrient profiles of struvite and brine. Struvite provides essential macronutrients (N, P, and Mg), while the brine serves as an excellent source of micronutrients, including Zn, Mn, B, Mo, and Fe, which are crucial for various metabolic processes in *A. platensis*. This synergistic nutrient provision likely contributes to the enhanced growth observed. Several studies demonstrated that Brine discharges from reverse osmosis plants include trace metals, such as iron (Fe), nickel (Ni), chromium (Cr), and molybdenum (Mo) (Omerspahic et al., 2022). The composition of the brine used in this study is presented in Table 1. When compared to brine compositions reported in other studies (Ahmed et al., 2001; Rivero-Falcón et al., 2023), brine used in the present study shows similarities in potassium and sodium levels, but differences in other elements. Notably, our brine contains lower levels of iron, manganese, and zinc, but detectable amounts of Mo and vanadium, which are not reported in the reference sources. Nitrogen content also varies significantly among the sources. It's important to note that brine composition can vary significantly depending on the location of the desalination plant, the seawater composition, and the technique used for desalination. This variability is evident in the diverse compositions reported in literature (as seen in Table 1). Consequently, these results, while promising, cannot be universally applied to microalgae media development based on brine without considering these compositional variations.

Recent studies have explored the use of struvite as an optimized nutrient source for microalgae cultivation. Struvite has been shown to support biomass productivity comparable to or higher than conventional media for various microalgae species (Chaoyu et al., 2017; Tang et al., 2023). It provides efficient nutrient utilization and can satisfy trace metal requirements (Davis et al., 2015). Moreover, the controlled nutrient release properties of struvite make it a valuable buffering nutrient source, with the potential to enhance the growth of microalgae (Tang et al., 2023). Experiments were carried out to assess the use of struvite as a primary nutrient source for cultivating two industrially relevant microalgae species: Nannochloropsis salina and Phaeodactylum tricornutum. These trials were conducted in both laboratory settings and outdoor pilot-scale raceways, spanning various seasonal conditions. The results demonstrated that media formulations containing either raw or refined struvite supported biomass production rates that were comparable to, or exceeded, those achieved with conventional growth media. In outdoor co-culture conditions, the peak biomass yield reached approximately 20 ± 4 g of ash-free dry weight per square meter per day. It demonstrates that these waste-derived nutrient sources can effectively support *A. platensis* growth, potentially as well as or better than conventional synthetic media. This finding supports the main thesis of using waste streams for sustainable microalgae cultivation (Davis et al., 2015). However, growth rates under BLT showed lower values compared to values reported in the literature (Islam et al., 2003; Usharani et al., 2012). This shows that blue light is less beneficial for the growth of *A. platensis* than yellow light. This can be attributed to the differences in the action spectra of photosynthesis (Jorgensen et al., 1987; Subramaniam et al., 1999). Although both light treatments had similar irradiances in the PAR region, the photosynthetic efficiency under yellow light is generally higher due to the action spectra of photosynthesis (Blanken et al., 2013). This difference can be attributed to several factors, a) Absorption efficiency where yellow light (570–590 nm) is more efficiently absorbed by chlorophyll a and phycobilins, the primary photosynthetic pigments in *A. platensis* (Markou et al., 2016). b) Photosystem balance where yellow light may provide a more balanced excitation of both photosystem I and II, potentially leading to more efficient electron transport and ATP and NADPH production, the essential molecules for carbon fixation and biomass accumulation, compared to blue light (Mohsenpour and Willoughby, 2013). This difference in photosynthetic response likely explains the increased biomass productivity observed under yellow light in the present study. Research on *A. platensis* cultivation under different light conditions reveals varying effects on biomass and pigment production. Red light generally promotes the highest biomass concentration and pigment production per culture volume (Tayebati et al., 2021). However, BLT results in the highest pigment content per dry weight, despite lower overall biomass (Tayebati et al., 2021). YLT enhances phycocyanin and allophycocyanin production in mixotrophic cultures (Yun et al., 2021). White light yields the highest biomass in mixotrophic conditions but lower pigment concentrations (Rizzo et al., 2015; Yun et al., 2021). Light quality significantly influences energy transfer processes within photosystems (Haury and Bogorad, 1977). Yellow light, in particular, enhances the energy transfer efficiency in phycobilisomes, which are connected to Photosystem II, as observed in both cyanobacteria and red algae (Akimoto et al., 2012; Biswas et al., 2024). This efficient energy transfer under yellow light is closely tied to the action spectra for photosynthesis, contributing to the overall photosynthetic efficiency and potentially explaining the higher biomass productivity observed in the present study.

While these results clearly demonstrate enhanced biomass and PC productivity under YLT compared to BLT, the underlying mechanisms require further investigation. The observed effects could be due to increased photosynthetic efficiency, photomorphogenic responses, or a combination of both. Photomorphogenesis, which involves gene induction in response to specific light qualities, can significantly influence the accumulation of bioactive compounds like PC. Previous studies have shown that blue or red light can induce photomorphogenic responses that enhance PC production (Pagels et al., 2020a; Pagels et al., 2020b). In order to distinguish between these processes, future experiments could adopt a photo biological strategy where photosynthesis is saturated under yellow light, followed by the application of low irradiances of blue or red light to evaluate their photomorphogenic effects. This approach would clarify the extent to which the observed increases in biomass and PC production under YLT are attributable to photosynthetic activity versus photo morphogenesis.

Overall, the growth of *A. platensis* in media enriched with brine (in addition to SAG micronutrients, BSI) was significantly higher than that in the SAG medium. However, the growth in media containing solely brine (BSIII, without micronutrients) was similar to the growth in the SAG medium. Previous reports showed that the effect of salinity on microalgae including *Arthrospira* varies. For example, optimal salinity levels for maximum biomass productivities of *Oocystis pusilla* were observed at around 3,000 ppm Total Dissolved Solids (TDS), resulting in significant increases in biomass yield compared to standard media. However, salinity levels beyond this optimum (e.g., 3,500 ppm TDS) led to marked reductions in productivity (Osman et al., 2023). Species-specific responses have been noted, with *A. maxima* showing continuous decreases in growth with increasing seawater levels, where final dry biomass decreased by 15% below the control (Tambiev et al., 2011). In addition, the growth of *A. platensis* completely stopped at seawater concentrations ranging between 40% and 60%. Liu et al. (2016) reported similar growth in *A. platensis* cultivated at low salinity (200 and 400 mM NaCl) to those obtained in the control Zarrouk medium, with slightly lower growth (−6%) at a salinity of 600 mM. A similar effect, decreasing growth and biomass yield, with increasing salinity, was reported in other studies (Polat et al., 2020; Tejada-Ruiz et al., 2020; Ghalhari et al., 2022).

The present salinity values that supported the highest growth in *A. platensis* were higher than those reported to inhibit the growth. One factor that might contribute to enhanced growth in the present study is the enrichment of brine with microalgal nutrients. Previous studies reported enhanced microalgal growth in brine. For instance, the growth of *C. vulgaris* enhanced when grown in 25% dilution of brine mixed with bold basal media (BBM), while brine dilutions of 30%–35% resulted in growth inhibition (Gilani et al., 2024). However, Tambiev et al. (2011) utilized sea salt sourced from Black Sea with Zarrouk’s medium at various volumetric ratios. Results confirmed the inhibitory effects of natural, pre-filtered, and autoclaved sea salt. Notably, the strain utilized in the present study (UTEX 2340) was not specifically previously evaluated for its high salt tolerance. These findings suggest that the strain exhibits a noteworthy tolerance to high salinity levels, which would play a crucial role in future applications.

Apart from the impact of struvite-brine-based media (SBBM) on growth, the present study aimed to evaluate if the SBBM potentially compromises C-PC enhancing effect of struvite recorded in the recent study (Beyer et al., 2023). Results confirmed that SBBM has no negative impact on C-PC yield. This may be related to the passive transfer of ammonia into the cell which may be independent of other minerals affecting active ion transfer across the cell membrane. As previously suggested (Beyer et al., 2023), higher C-PC is correlated to a nitrogen-sparing effect associated with ammonia fraction in struvite. The present study also demonstrated that SBBM enhanced C-PC production, especially during the exponential growth phase. This has an important industrial value since the harvesting time of biomass and C-PC will be towards the end of this phase. C-PC measurements were done mainly to confirm that there is no negative effect on C-PC associated with brine in the media and this was confirmed. Recent studies on *A. platensis* cultivation have revealed strategies to enhance both biomass and C-phycocyanin (C-PC) production. Light intensity significantly affects growth and product formation, with lower intensities favoring C-PC production and higher intensities promoting extracellular polymeric substances (Dejsungkranont et al., 2017). Optimal light intensities for cell growth (282 μmol m^−2^ s^−1^) and phycocyanin synthesis (137 μmol m^−2^ s^−1^) differ, with light attenuation impacting intracellular phycocyanin content (del Rio-Chanona et al., 2015). Research on light quality and intensity effects on microalgal cultivation has shown promising results for enhancing biomass and phycocyanin production. White light in mixotrophic conditions yielded the highest biomass concentration for *A. platensis* (Chainapong et al., 2012). However, yellow and red lights in mixotrophic cultures produced the highest phycocyanin content (Chainapong et al., 2012). White light generally results in higher biomass and phycocyanin production compared to green and blue light (Jung et al., 2022). Moreover, the overall yield was not improved under BLT due to the lower growth rate of *A. platensis*. These results highlight the complex interplay between nutrient availability, light conditions, and metabolite production in *A. platensis*.

The aforementioned findings have significant implications for sustainable *A. platensis* cultivation, demonstrating that simplified, potentially more cost-effective and environmentally friendly media based on brine and struvite can support and even enhance C-PC production, particularly when combined with appropriate light conditions. The success of BS III, in particular, highlights its potential for developing highly efficient minimal growth medium for industrial-scale production of this valuable cyanophyte. The data clearly shows that yellow-white light consistently leads to higher biomass and C-PC productivity across all media types compared to the blue-white light. Under yellow-white light, the brine-struvite-based media (BS I, II, and III) outperformed the standard SAG medium, with BS I showing the highest C-PC productivity, followed closely by BS III. In contrast, under blue-white light, C-PC productivity was generally lower and more consistent across media types, with BS III showing a slight advantage. The standard SAG medium consistently yielded the lowest C-PC productivity under both light conditions. These results suggest that using brine-struvite-based media, particularly BS I or BS III, in combination with yellow-white light, could significantly enhance C-PC production in *A. platensis* cultures compared to the standard media and lighting conditions.

## 5 Conclusion

The present study marks a significant milestone by demonstrating that *A. platensis* can be cultivated using local waste streams, i.e., combined brine and struvite, to effectively substitute mineral-based synthetic growth mediums. Results not only offer a solution for valorizing and reducing waste streams from the agricultural industry but also present an innovative approach for the emerging hydrogen energy sector to address brine disposal challenges in the coming decades. Moreover, the novel media tested in this study provides the microalgal industry with a cost-effective and highly sustainable alternative. The incorporation of brine and struvite as nutrient sources resulted in enhanced growth rates, showing the potential synergistic effects between natural and synthetic components. Despite the variability in brine composition, the observed growth enhancements confirmed the adaptability of the cultivated *A. platensis* to diverse environmental conditions. Furthermore, the study confirmed that the use of SBBM does not influence the production of C-PC as a valuable pigment with many industrial applications, suggesting the need of further biotechnological exploitation. These findings not only expand the current understanding of microalgae cultivation, but also highlight the potential for integrating waste valorization and sustainable resource utilization in bioprocessing industries. The broader implications of this research are far-reaching, potentially revolutionizing multiple sectors including circular economy practices, sustainable agriculture, clean energy transition, food and nutrition security, and biotechnology. Future research directions could include scaling-up the cultivation process, investigating other microalgal species, exploring geographical variations, developing automated optimization systems, and conducting comprehensive life cycle analyses. By addressing these areas, future studies can build upon this foundational work, further advancing the fields of sustainable biotechnology and circular economy practices.

## Data Availability

The original contributions presented in the study are included in the article/Supplementary Material, further inquiries can be directed to the corresponding author.
