# Supplementary material for: Potential of a novel brine-struvite-based growth medium for sustainable biomass and phycocyanin production by Arthrospira platensis
Source: Front Bioeng Biotechnol. 2024 Oct 2;12:1466978. doi: 10.3389/fbioe.2024.1466978 (PMC11479874; doi:10.3389/fbioe.2024.1466978)
Supplement: Supplementary file 1 [file DataSheet1.PDF]

Supplementary Material

Supplementary Table S1: Tukey's Honestly Significant Difference (HSD) test, comparing the means of maximum generation time [T2max] calculated by Gompertz fit at 480 nm (A) and 700 nm (B) for each culture medium. At a P-value lower than 0.05, the means between the maximum generation times of 480 nm and 700 nm are significantly different.

A

TUKEY	MeanDiff	q Value	P-value
BS I SAG	0,03	5,3	0,023
BS II SAG	-0,03	5,1	0,028
BS II BS I	-0,07	10,4	0,000
BS III SAG	0,04	6,4	0,009
BS III BS I	0,01	1,1	0,875
BS III BS II	0,07	11,5	0,000

B

TUKEY	MeanDiff	q Value	P-value
BS I SAG	0,05	7,3	0,004
BS II SAG	-0,03	5,1	0,030
BS II BS I	-0,08	12,4	0,000
BS III SAG	0,05	7,1	0,004
BS III BS I	0,00	0,2	0,999
BS III BS II	0,08	12,2	0,000

Supplementary Table S2: Tukey's Honestly Significant Difference (HSD) test, calculated via two sample t-testing between the means of maximum generation time [T2max] calculated by Linear Regression at 480 nm (A) and at 700 nm (B) for each culture medium. At a P-value lower than 0.05, the means between the maximum generation times of 480 nm and 700 nm are significantly different.

A

TUKEY	MeanDiff	q Value	P-value
BS I SAG	0,02	4,6	0,048
BS II SAG	-0,03	8,5	0,001
BS II BS I	-0,05	13,1	0,000
BS III SAG	0,01	1,5	0,737
BS III BS I	-0,01	3,1	0,205
BS III BS II	0,04	10,0	0,000

B

TUKEY	MeanDiff	q Value	P-value
BS I SAG	0,02	6,0	0,012
BS II SAG	-0,03	8,4	0,002
BS II BS I	-0,06	14,4	0,000
BS III SAG	0,00	1,2	0,837
BS III BS I	-0,02	4,8	0,038
BS III BS II	0,04	9,6	0,001

Supplementary Table S3: Tukey's Honestly Significant Difference (HSD) test, calculated via two sample t-testing between the means of C-PC productivity. The data was calculated from the interval of early exponential phase [day 0 to day 8 (A)] to late exponential phase [day 0 to day 14 (B)] during the cultivation period.

A

TUKEY	q Value	P-value	Alpha
BSI SAG	13.85	0.00	0.05
BSII SAG	11.97	0.00	0.05
BSII BSI	1.87	0.57	0.05
BSIII SAG	13.49	0.00	0.05
BSIII BSI	0.35	0.99	0.05
BSIII BSII	1.52	0.71	0.05

B

TUKEY	q Value	P-value	Alpha
BSI SAG	0.90	0.92	0.05
BSII SAG	1.06	0.87	0.05
BSII BSI	0.17	1.00	0.05
BSIII SAG	3.80	0.10	0.05
BSIII BSI	2.90	0.25	0.05
BSIII BSII	2.73	0.29	0.05

Supplementary Table S4: Shown are the values of the c-phycocyanin production per g of dry weight per day, calculated from the values of the biomass and c-phycocyanin productivity. The values represent the c-phycocyanin increase during the exponential growth phase from day 0 to day 8 for the yellow-white light cultivation conditions [A] and from day 0 to day 7 for the blue-white light cultivation conditions [B] for the cultivation with different culture media [SAG, BSI, BSII and BSIII]. Additionally, the standard error and standard derivation are shown, as well as the increase of each parameter in relation to the control group [SAG].

Lights	Parameter	*CPC production per g DW per day*			
	Medium	SAG	BSI	BSII	BSIII
	Unit	[mg g^{-1} d^{-1}]	[mg g^{-1} d^{-1}]	[mg g^{-1} d^{-1}]	[mg g^{-1} d^{-1}]
Yellow-white light	*Mean*	2.97	12.70	12.45	12.74
	SE [±]	0.09	0.52	0.30	0.85
	SD	0.16	0.91	0.52	1.48
	Increase to SAG (-) [%]	0	327.8	319.3	328.9
	**B**				
			BSI	BSII	BSIII
					[mg g^{-1} d^{-1}]
Blue-white light	*Mean*	15.07	13.39	19.08	14.28
	SE [±]	2.75	1.17	1.49	2.56
	SD	4.75	2.03	2.58	4.43
	Increase to SAG (-) [%]	0	-11.1	26.6	-5.2

Supplementary Table S5. Tukey's Honestly Significant Difference (HSD) test, calculated via two sample t-testing between the means of C-PC-content yield (mg $g^{-1}d^{-1}$) for the cultures grown in different culture media (SAG, BS I, BS II, BS III] under yellow-white light (YLT), showing the significant differences values between media in blue-white light (BLT) and SAG of YLT.

YLT

TUKEY	MeanDiff	q-Value	*P*-values
BS I -SAG	9.73	15.13	<0.001
BS II - SAG	9.48	14.74	<0.001
BS II - BS I	-0.25	0.39	0.992
BS III – SAG	9.77	15.18	<0.001
BS III - BS I	0.03	0.05	1
BS III - BS II	0.29	0.45	0.988

BLT-YLT

TUKEY	MeanDiff	q-Value	*P*-values
SAG (BLT) - SAG (YLT)	12.10	6.45	0.006
BS I (BLT) - SAG (YLT)	10.42	5.56	0.021
BS II (BLT) - SAG (YLT)	16.11	8.59	0.000
BS III (BLT) - SAG (YLT)	11.31	6.03	0.011

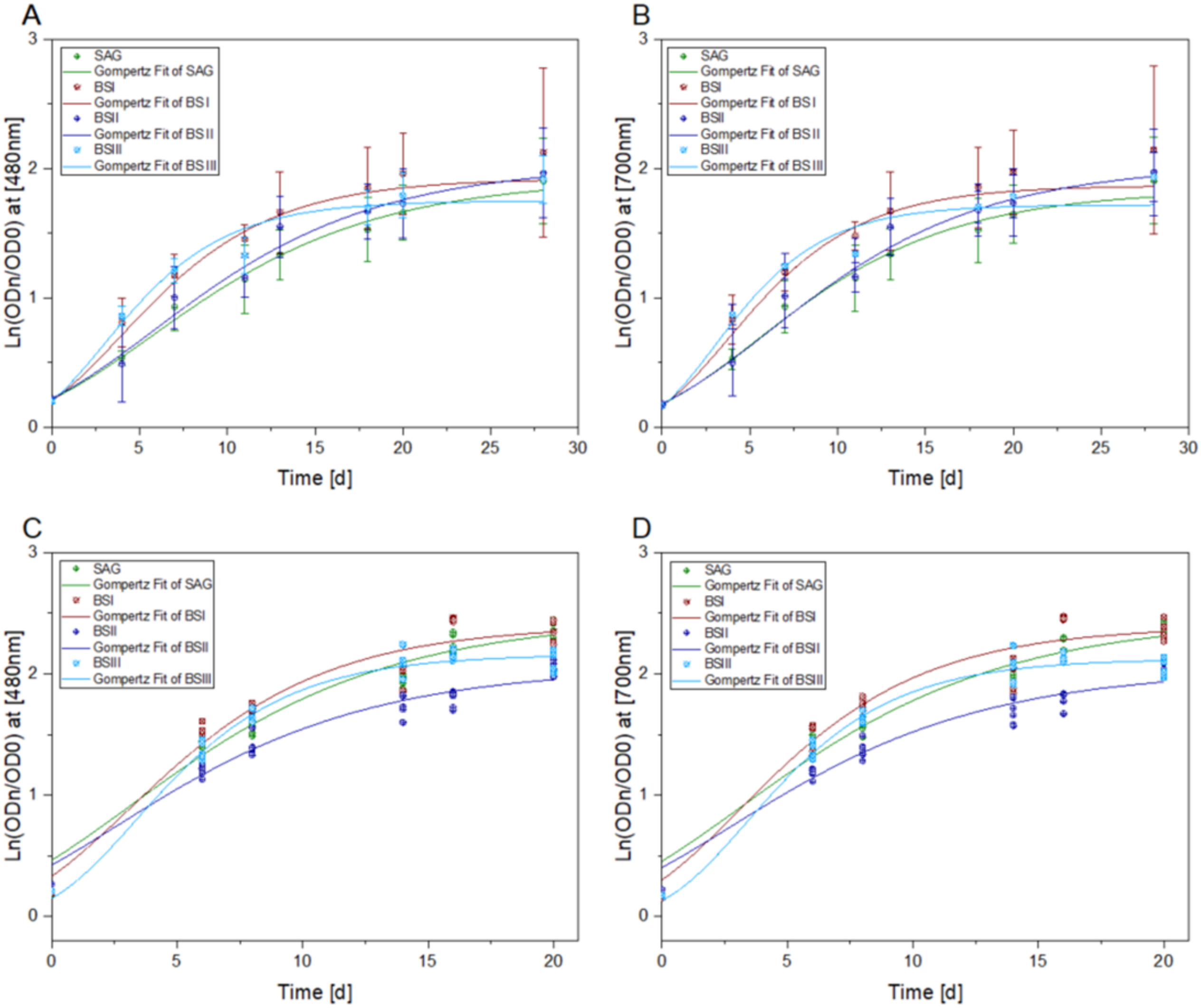

Figure S1. Growth curves of *A. platensis* cultures grown in four culture media [SAG, BS I, BS II, BS III] under yellow-white light (A, B) and blue-white light (C, D). Each data point shows the mean of the values derived from six absorbance measurements at 480 nm (A, C) and 700 nm (B,D) for each time point. n = 66 per treatment, F- and *P*-values for dataset of A: F = 0.59; *P* = 0.62, F- and *P*-values for dataset of B: F = 0.57 *P* = 0.64. F- and *P*-values for dataset of C: F = 0.59; *P* = 0.62, F- and *P*-values for dataset of D: F = 0.57 *P* = 0.64.
